# A Case of Combined Hepatocellular‐Cholangiocarcinoma Diagnosis and Treatment With Literature Review

**DOI:** 10.1002/ccr3.70652

**Published:** 2025-08-13

**Authors:** Xuliang Chen, Hengkai Zhu, Liyan Tao, Shusen Zheng, Hua Guo

**Affiliations:** ^1^ Department of Hepatobiliary and Pancreatic Surgery, Key Laboratory of Artificial Organs and Computational Medicine in Zhejiang Province, Shulan (Hangzhou) Hospital, Shulan International Medical College Zhejiang Shuren University Hangzhou P. R. China

**Keywords:** adult liver cancer, cancer reports, chemoembolization, drug therapy, hepatectomy, radiotherapy

## Abstract

This report presents a rare case of a 43‐year‐old man with unresectable combined hepatocellular‐cholangiocarcinoma who, after undergoing transarterial chemoembolization, systemic chemotherapy, and stereotactic body radiation therapy, accepted curative resection and obtained an overall survival of 25 months to date. The case highlights the need for adaptable treatment regimens due to the tumor's components and underscores the importance of surgery for long‐term success.

**Summary**

- cHCC‐CCA comprises both HCC and ICC, which leads to refractory therapeutic responses due to the complex biological behaviors of these two histological components.
- Therefore, treatment strategies must be correspondingly revised based on serial assessments of tumor responses.

**Abbreviations**
AFP: alpha‐fetoprotein
CA19‐9: carbohydrate antigen 19–9
cHCC‐CCA: combined hepatocellular‐cholangiocarcinoma
CK19: cytokeratin‐19
CT: computed tomography
CTP: Child‐Turcotte‐Pugh
DCR: disease control rate
DFS: disease‐free survival
d‐TACE: drug‐eluting transarterial chemoembolization
GEMOX: gemcitabine plus oxaliplatin
GP: gemcitabine plus platinum‐based agents
GPC‐3: glypican‐3
HBsAg: hepatitis B surface antigen
HBV: hepatitis B virus
HCC: hepatocellular carcinoma
ICC: intrahepatic cholangiocarcinoma
MDT: multi‐disciplinary treatment
MWA: microwave ablation
NCCN: National Comprehensive Cancer Network
ORR: objective response rate
OS: overall survival
PD‐1: programmed cell death protein 1
PVTT: portal vein tumor thrombus
SBRT: stereotactic body radiation therapy
SD: stable disease
TACE: transarterial chemoembolization

## 1 Introduction

Combined hepatocellular‐cholangiocarcinoma (cHCC‐CCA) represents a rare form of primary liver tumor, characterized by the coexistence of hepatocellular carcinoma (HCC) and intrahepatic cholangiocarcinoma (ICC) histological features shown in pathological examination. Although cHCC‐CCA constitutes a low percentage among primary liver cancers [1, 2, 3, 4], it is associated with a poor prognosis and ranks second in mortality worldwide [5]. Recent advancements in imaging and pathological diagnostic techniques have led to a significant increase in the detection rate of cHCC‐CCA. It is generally accepted that cHCC‐CCA is mostly observed in male patients with cirrhosis or chronic liver disease [2, 6]. Nonetheless, therapeutic approaches for this malignancy remain limited, with a paucity of well‐defined consensus and clinical guidelines. In our clinical practice, we treated a typical case of cHCC‐CCA. The patient presented with an advanced‐stage tumor and a high intrahepatic tumor burden. By integrating recommendations from the limited guidelines and multidisciplinary therapeutic strategies, the patient ultimately underwent right hemihepatectomy, and we achieved a favorable clinical effect. The aim of this case report is to describe the treatment process and provide practical guidance for similar cases.

## 2 Case Presentation

**FIGURE 1:**
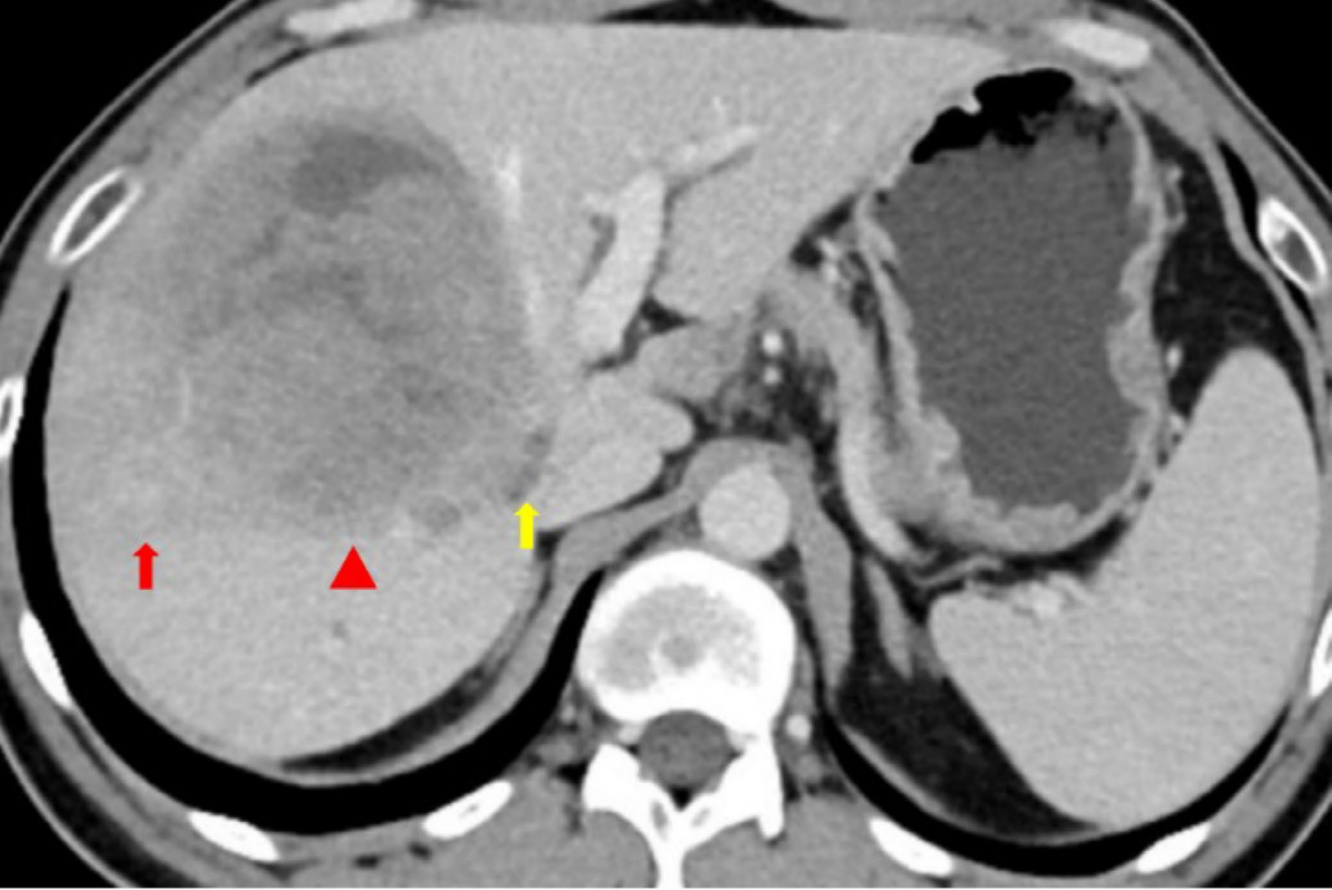
Contrast‐enhanced CT scan reveals a right hepatic mass (red triangle), with a satellite nodule (red arrow) and a tumor thrombus in the right hepatic vein (yellow arrow).

**FIGURE 2:**
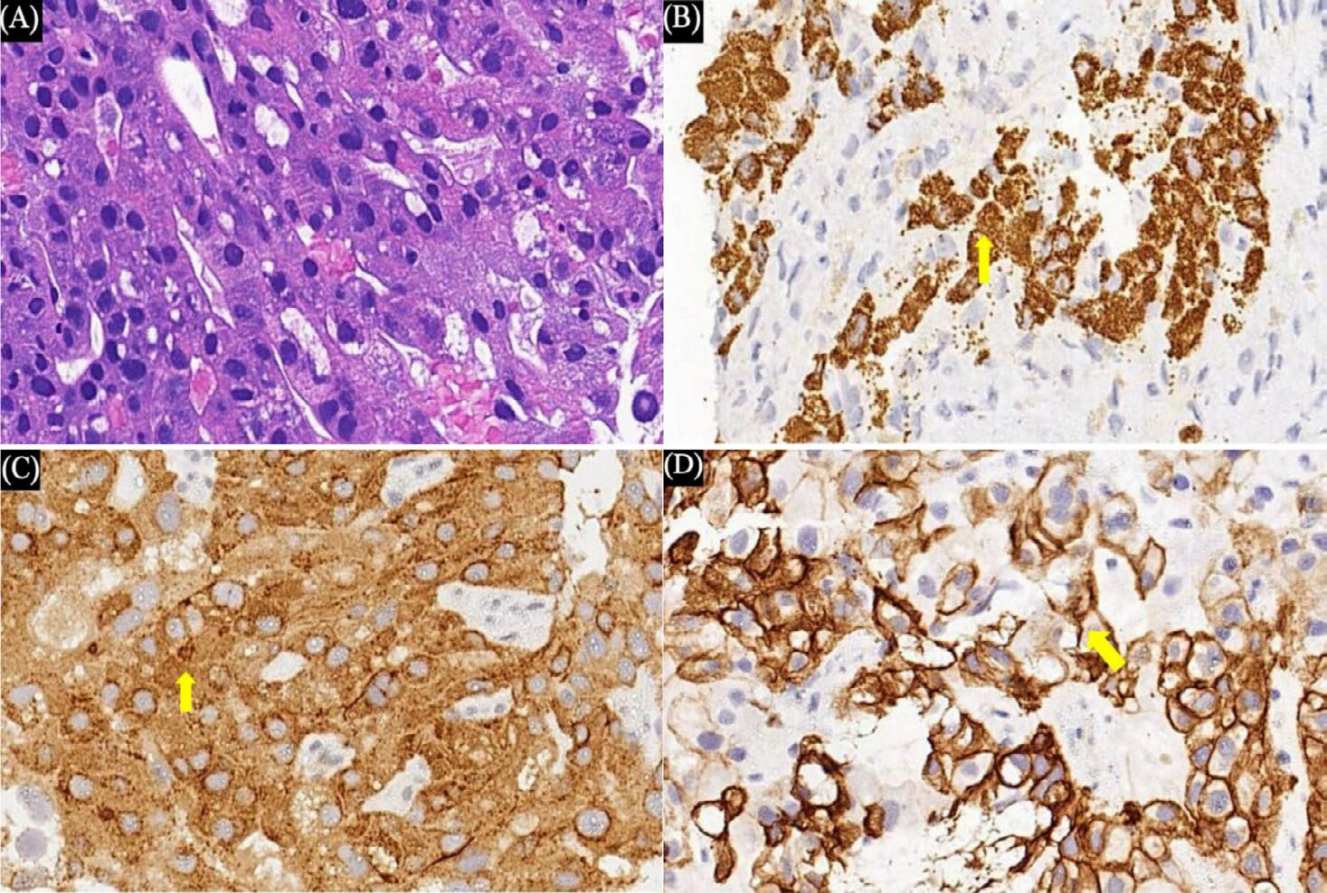
(A) Hematoxylin and eosin stain; original magnification, ×400. (B) Positive cytoplasmic expression of HepPar‐1 (yellow arrow). Immunohistochemical stain; original magnification, ×400. (C) Strong nuclear and cytoplasmic positivity for GPC‐3 (yellow arrow). Immunohistochemical stain; original magnification, ×400. (D) Diffuse membranous staining of CK19 (yellow arrow). Immunohistochemical stain; original magnification, ×400.

A 43‐year‐old male underwent computed tomography (CT) imaging, which revealed a right hepatic mass 9.6 × 8.2 × 8.7 cm approximately, with a satellite nodule and tumor thrombus in the right hepatic vein (Figure 1). Laboratory tests showed: positive Hepatitis B surface antigen (HBsAg), elevated Alpha‐fetoprotein (AFP) (402.4 ng/mL) and Carbohydrate antigen 19–9 (CA19‐9) (1258.8 U/mL). Liver biopsy demonstrated biphenotypic cHCC‐CCA (Figure 2). Immunohistochemical analysis revealed positive expression of HepPar‐1, Glypican‐3 (GPC‐3), and Cytokeratin‐19 (CK19) within the tumor field, and the diagnosis of biphenotypic cHCC‐CCA was established.

## 3 Investigations and Treatment

**FIGURE 3:**
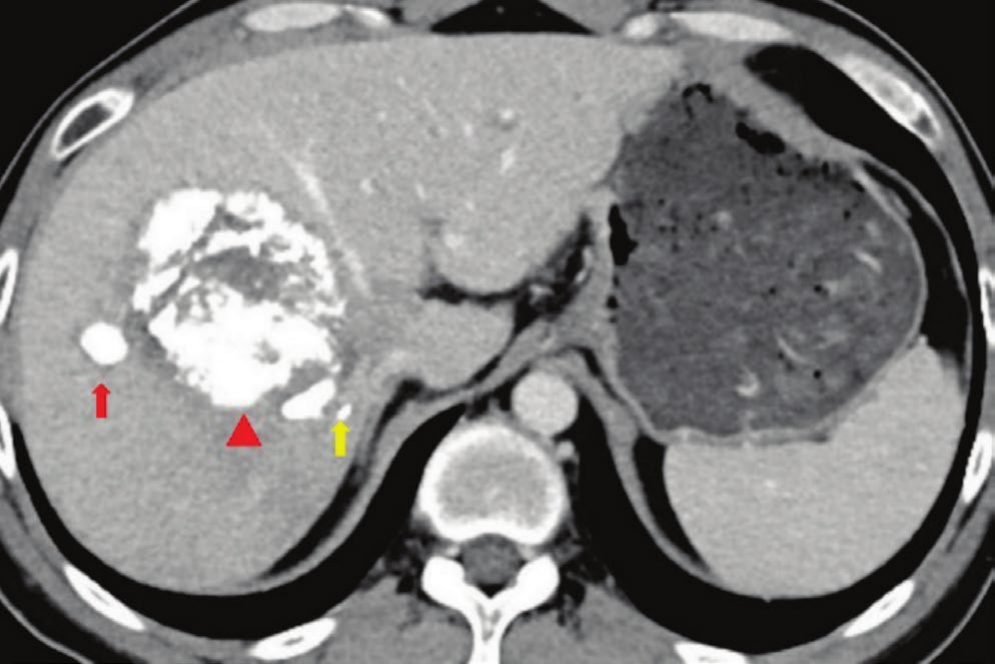
CT scan reveals a hepatic mass (red triangle) with complete lipiodol deposition in the satellite nodule (red arrow) and the smaller tumor thrombus in the right hepatic vein (yellow arrow).

The patient underwent drug‐eluting transarterial chemoembolization (d‐TACE) soon. One month later, AFP and CA19‐9 decreased to 105.4 ng/mL and 595 U/mL, respectively. One month later, the patient underwent three cycles of gemcitabine plus oxaliplatin (GEMOX) regimen. Two months later, CA19‐9 decreased to 187.8 U/mL, whereas AFP increased to 389.6 ng/mL. The tumor response was assessed as stable disease (SD) by imaging examinations. Three months later, the patient underwent TACE and stereotactic body radiation therapy (SBRT) for the tumor thrombus in the right hepatic vein. Subsequently, AFP and CA19‐9 decreased to 91.5 ng/mL and 50.3 U/mL, respectively. Four months later, the patient underwent a fourth cycle of GEMOX regimen, resulting in further decrease of AFP (68 ng/mL) and CA19‐9 (42.9 U/mL). CT scan revealed no enhancement in the primary tumor and the satellite nodule, along with a smaller size of the tumor thrombus in the right hepatic vein (Figure 3). In the fifth month, the patient underwent right hemihepatectomy after MDT. Pathological examination revealed complete necrotic cHCC‐CCA tissue, about 8 × 8 × 7.5 cm. Two weeks after hepatectomy, the patient underwent the first cycle of adjuvant GEMOX regimen, which was subsequently discontinued due to grade‐3 myelosuppression.

## 4 Outcomes and Follow‐Up

**FIGURE 4:**
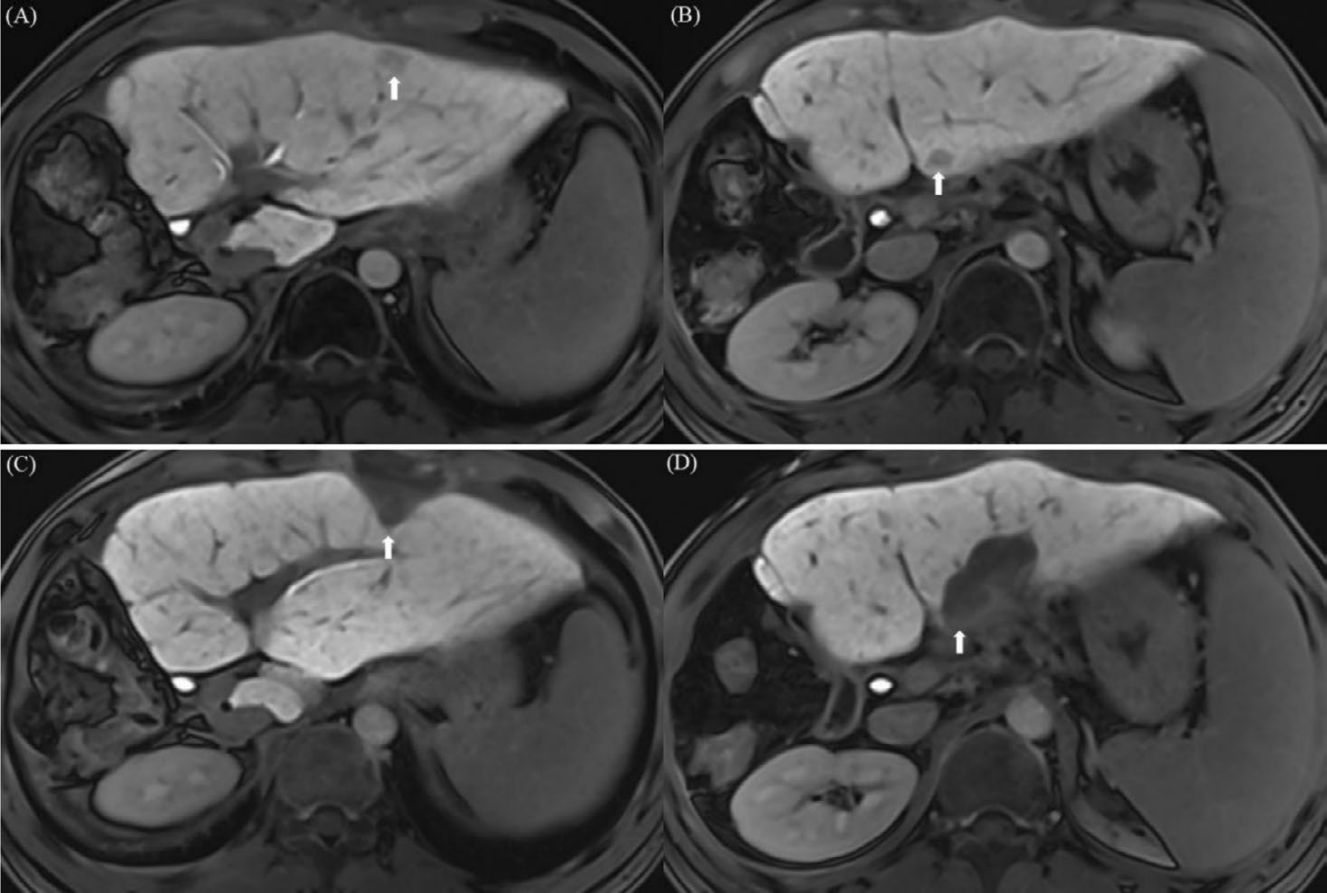
(A and B) Gd‐EOB‐DTPA‐enhanced MRI scans reveal two de novo nodules (white arrow) in the left hepatic lobe. (C and D) After MWA, imaging examinations reveal the complete ablation of both nodules (white arrow).

**FIGURE 5:**
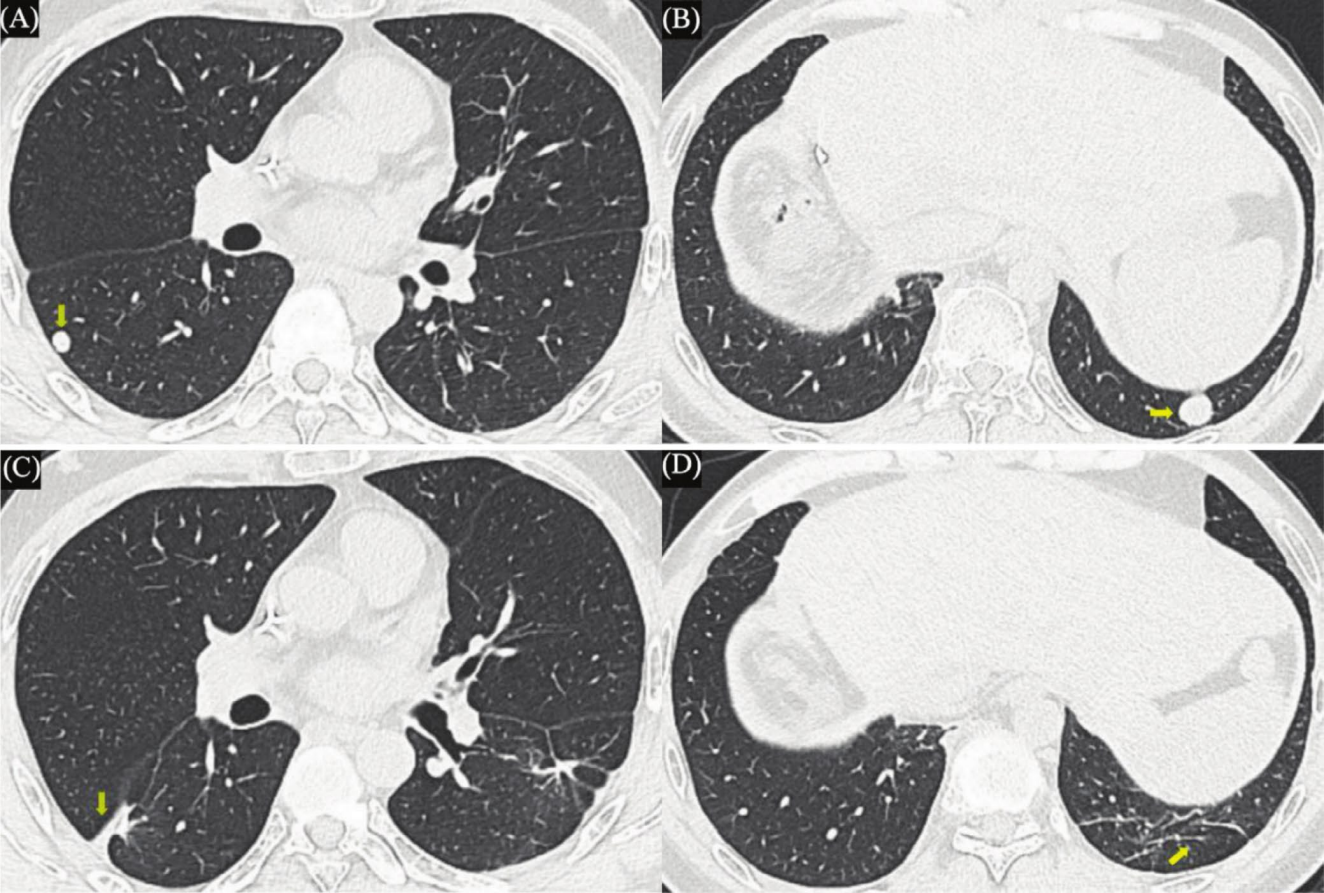
(A and B) CT scans reveal two de novo lung nodules (yellow arrow). (C and D) CT scans reveal the complete resection of two de novo lung nodules (yellow arrow).

**FIGURE 6:**
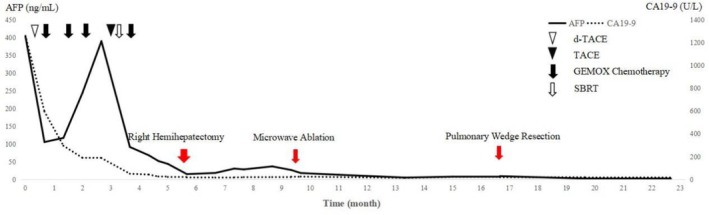
Progressive decline of serum AFP and CA19‐9 to normal ranges after multimodal therapies including TACE, chemotherapy, SBRT and resection.

Five months after the hepatectomy, contrast‐enhanced ultrasound revealed two de novo nodules in the left hepatic lobe, accompanied by a mild increase of AFP and CA19‐9, and tumor recurrence was considered initially. Then, the patient underwent microwave ablation (MWA) (Figure 4) and 6‐month disease‐free survival (DFS) was achieved. Seven months after MWA, the CT scan revealed two de novo pulmonary nodules (Figure 5), considered as pulmonary metastases of cHCC‐CCA. The patient subsequently underwent a video‐assisted thoracoscopic wedge resection, and pathological examination of metastatic cHCC‐CCA was confirmed. After lung wedge resection, the patient achieved a 7‐month DFS again. After the above series of treatments, the patient has survived 25 months, and close follow‐up has remained ongoing. A chart of AFP and CA19‐9 during the course is provided (Figure 6).

## 5 Discussion

Primary liver cancer is the seventh most common malignancy in terms of incidence and ranks second in mortality worldwide [5]. cHCC‐CCA constitutes 1%–10% of primary liver cancers and is mostly observed in male patients with cirrhosis or chronic liver disease [2, 6]. The etiological profiles and risk factors for cHCC‐CCA vary significantly across different geographical regions [7, 8]. For example, in Asian countries, the condition is mostly caused by hepatitis virus and liver cirrhosis, often with high AFP levels. In contrast, in Western countries, cHCC‐CCA is more commonly linked to heavy alcohol use and nonalcoholic liver diseases [9, 10]. In this case, the patient was a male with a history of hepatitis B virus (HBV) infection, significantly high AFP and CA19‐9 levels, a large right hepatic mass, a satellite nodule, and tumor thrombus in the right hepatic vein. Liver biopsy confirmed biphenotypic cHCC‐CCA, and the HBV‐associated cHCC‐CCA diagnosis was established.

Currently, there are no established standard therapies for cHCC‐CCA [10, 11]. Surgical resection remains the only potentially curative option, but it applies to only a minority of patients [12]. According to the National Comprehensive Cancer Network (NCCN) guidelines, patients with Child‐Turcotte‐Pugh (CTP) class A or highly selected patients with CTP class B, suitable tumor location, adequate liver reserve, suitable liver remnant, and without portal hypertension can undergo surgical resection. Meanwhile, patients with advanced‐stage liver cancer may undergo reassessment for surgical resection after conversion therapy [13]. Non‐surgical therapies are mainly derived from those for HCC and ICC. TACE is a key treatment for intermediate‐to‐advanced HCC, but its efficacy in cHCC‐CCA patients is less favorable than in HCC. A propensity score‐matched analysis conducted by Mukund et al. [14], which included 13 cases of cHCC‐CCA and 101 cases of HCC treated with TACE‐based locoregional therapy, revealed that patients with cHCC‐CCA had poorer prognosis than those with HCC in DFS (1.5 months vs. 7.5 months), overall survival (OS) (12 months vs. 28 months) and objective response rate (ORR) (25% vs. 91%). The findings reported by Huang et al. [15] are largely in agreement with those of Mukund et al. Additionally, a retrospective study of 50 cHCC‐CCA patients treated with TACE found 85% (34/40) of hypervascular tumors responded, versus 10% (1/10) for hypovascular tumors [16]. These results indicate tumor vascularity is a key factor influencing the efficacy of TACE in cHCC‐CCA. Nikolaos et al. [17] evaluated gemcitabine plus platinum (cisplatin or oxaliplatin) as first‐line therapy in advanced cHCC‐CCA, reporting a median OS of 10.2–16.2 months and a median PFS of 3.0–9.0 months. Kobayashi et al. [18] found similar results. Nikolaos et al. [17] also showed gemcitabine plus platinum (GP) achieved ORR 24.3% versus 15.4% and disease control rate (DCR) 78.4% versus 38.5% compared with gemcitabine alone or fluorouracil. In cases with vascular invasion, SBRT also represents an effective therapy. A systematic review showed SBRT followed by TACE resulted in improved OS and ORR compared with TACE alone in HCC patients with portal vein tumor thrombus (PVTT) [19]. This finding suggests PVTT may exhibit radiosensitization to SBRT, and SBRT could enhance tumor vascularity, thereby improving the therapeutic efficacy of TACE. Additionally, Kim et al. [20] reported that SBRT followed by TACE improved OS to 15.3 months versus 7.5 months with TACE alone in cases of massive HCC.

In this case, the patient presented with an advanced‐stage tumor and a high intrahepatic tumor burden. Initial treatment combined d‐TACE and GEMOX regimen to achieve rapid tumor control. Two months later, AFP decreased mildly while CA19‐9 decreased significantly, suggesting a poor response in the HCC component and prompting treatment adjustment. Subsequently, combined TACE and SBRT induced significant necrosis of both the primary tumor and the satellite nodule, as well as reduction of the hepatic vein tumor thrombus. After MDT, the patient was found suitable for radical resection. Despite the recurrence of intrahepatic nodules and the development of pulmonary metastasis after hepatectomy, the patient survived 25 months through MWA and wedge resection of the lung. Close follow‐up goes on.

## 6 Conclusion

cHCC‐CCA is a rare primary liver carcinoma, with hepatitis virus infections and liver cirrhosis as main risk factors. cHCC‐CCA comprises both HCC and ICC, which leads to refractory therapeutic responses due to the complex biological behaviors of these two histological components. Therefore, treatment strategies must be correspondingly revised based on serial assessments of tumor responses. In patients with advanced cHCC‐CCA, we suggest that therapies such as TACE, systemic chemotherapy, and SBRT should achieve tumor downstaging and enable radical resection further. Furthermore, it is important to emphasize that each therapeutic decision was made by Multi‐Disciplinary Treatment (MDT); long‐term and close follow‐up is necessary.

## Author Contributions

**Xuliang Chen:** data curation, formal analysis, investigation, methodology, visualization, writing – original draft. **Hengkai Zhu:** funding acquisition, project administration, writing – review and editing. **Liyan Tao:** resources, software, writing – review and editing. **Shusen Zheng:** supervision, writing – review and editing. **Hua Guo:** conceptualization, validation, writing – review and editing.

## Disclosure

*Permission to reproduce material from other sources*: All reproduced images appearing in the manuscripts have been consented to by the patient, the leading doctor, and the hospital.

## Ethics Statement

This study was approved by the Ethics Committee of Shulan (Hangzhou) Hospital. The cases reported in this article have been approved by the patient, the leading doctor, and the hospital.

## Consent

Written informed consent was obtained from the relative of the patient for publication of this case report and any accompanying images. A copy of the written consent is available for review by the Editor of this journal.

## Conflicts of Interest

The authors declare no conflicts of interest.

## Data Availability

The data used and analyzed in this case report are available from the corresponding authors on reasonable request.
